# Lomitapide, a Microsomal Triglyceride Transfer Protein Inhibitor, in Homozygous Familial Hypercholesterolemia: A Systematic Review and Meta-Analysis of Efficacy and Safety

**DOI:** 10.1007/s10557-025-07764-4

**Published:** 2025-08-26

**Authors:** Mohamed Nasser, Hazem E. Mohammed, Mohamed E. Haseeb, Mohamed Khalafalla Darwish, George Hanen, Nada A. Abdelaziz, Anas Hussien Heiba, Abdelrahman Shata, Mohamed Salem Abdelkader

**Affiliations:** 1 Faculty of Medicine, Minia University, Minia, Egypt; 2 Faculty of Medicine, Assiut University, Assiut, Egypt; 3 Faculty of Medicine, Horus University-Egypt, New Damietta, Egypt; 4 Medical Research Group of Egypt (MRGE), Negida Academy, Cairo, Egypt

**Keywords:** Homozygous Familial Hypercholesterolemia, HoFH, Lomitapide, Microsomal triglyceride transfer protein inhibitor, Systematic review, Meta-analysis

## Abstract

**Purpose:**

Homozygous Familial Hypercholesterolemia (HoFH) is a rare and life-threatening genetic disorder characterized by elevated low-density lipoprotein cholesterol (LDL-C) and early-onset atherosclerotic cardiovascular disease. Lomitapide, a microsomal triglyceride transfer protein (MTP) inhibitor, decreases LDL-C independent of LDL receptor function, providing an alternative treatment in this population. We aimed to evaluate the efficacy and safety of lomitapide in patients with HoFH through a systematic review and meta-analysis of available clinical evidence.

**Methods:**

A comprehensive search was conducted in PubMed, Scopus, and Web of Science through March 2025. Observational studies and clinical trials reporting on lipid profile changes and safety outcomes in HoFH patients receiving lomitapide were included. Outcomes were pooled using random-effects models, and heterogeneity was assessed using the I^2^ statistic.

**Results:**

Eight studies comprising both adult and pediatric patients (n = 209) were included. Lomitapide significantly reduced LDL-C levels by 49.27%, total cholesterol by 46.05%, and apolipoprotein B by 51.01%. Reductions were also observed in triglycerides, VLDL-C, and non–HDL-C. HDL-C remained relatively unchanged. Adverse events were mostly gastrointestinal, with a 14% discontinuation rate. The overall quality of studies ranged from fair to good.

**Conclusions:**

Lomitapide demonstrates substantial efficacy in reducing LDL-C and other atherogenic lipids in HoFH patients, with an acceptable safety profile. These findings support its role as an adjunctive therapy in this population, though further randomized controlled trials are warranted to validate long-term safety and effectiveness.

**Supplementary Information:**

The online version contains supplementary material available at 10.1007/s10557-025-07764-4.

## Background

Homozygous Familial Hypercholesterolemia (HoFH) is a life-threatening autosomal dominant hereditary disease characterized primarily by extremely high serum levels of low-density lipoprotein cholesterol (LDL-C) (> 500 mg/dL) and accelerated development of atherosclerotic cardiovascular disease (ASCVD). Affecting approximately 1 in 300,000 individuals globally, with a higher prevalence in areas of consanguinity such as Southeast Asia and the Western Pacific, the disease results from gene mutations in the LDL receptor (LDLR) pathway that cause a loss of receptor function and impair the clearance of LDL from circulation [1, 2].

Patients with HoFH often develop premature atherosclerosis, presenting with manifestations such as xanthomas, corneal arcus, and cardiovascular complications—including myocardial infarctions, aortic stenosis, and coronary artery disease—before the age of twenty, leading to markedly reduced life expectancy [3]. The primary treatment approach involves lipid-lowering therapies such as high-intensity statins, ezetimibe, and proprotein convertase subtilisin/kexin type 9 (PCSK9) inhibitors, aiming to control hypercholesterolemia, prevent ASCVD, and reduce serum LDL-C levels in adults with HoFH to below 100 mg/dL, as recommended by the European Atherosclerosis Society [1, 4–8]. However, these therapies rely on residual LDL receptor function, which is often severely impaired in HoFH patients. Consequently, achieving target LDL-C levels remains challenging. Evinacumab which is an inhibitor of ANGPTL3 has become an acceptable substitution as mentioned in European Atherosclerosis Society (EAS) guidelines [9]. In phase 3 of, randomized, placebo-controlled trial (ELIPSE HoFH), its addition to the maximum doses of lipid-lowering therapy resulted in a 47.1% reduction in LDL cholesterol from baseline, compared to a 1.9% increase in the placebo group [10]. However, its limitations include some serious side effect as urosepsis, suicidal attempts and elevated liver enzymes as reported by Frederick J et al. Additionally, it is only licensed for HoHF patients aged at least 12 years so it’s restricted for children younger than that which limits itsapplication. In addition to requirement for intravenous administration every four weeks and as a safety indication a concerning 29.6% drop from the baseline in HDL cholesterol levels has been noted and identified [9, 10]. Another treatment option is lipoprotein apheresis, which removes LDL-C from circulation through adsorption. However, its use is constrained by logistical challenges, cost barriers, availability issues, and the need for regular sessions due to rapid re-accumulation of LDL-C in the bloodstream [11]. Liver transplantation has been considered a curative treatment since it restores functional LDL receptors.

Lomitapide, a microsomal triglyceride transfer protein (MTP) inhibitor, prevents hepatic transfer of triglycerides into apolipoprotein B. This mechanism reduces the secretion of very-low-density lipoprotein (VLDL), an LDL-C precursor, from the liver. As a lipid-lowering agent independent of LDL receptor pathways, lomitapide is particularly effective for HoFH patients. Currently used as an alternative or adjunctive therapy, it has demonstrated promising effects in reducing LDL-C levels by approximately 50% in adults [11–14], with recent studies showing efficacy in pediatric populations as well [15]. Despite these positive results, further studies are needed to assess and confirm the long-term safety and efficacy of lomitapide in reducing LDL-C alongside other serum lipids across diverse HoFH patient groups, with associated gastrointestinal and hepatic dysfunction adverse effects being reported. The aim of this meta-analysis was to provide a comprehensive review and assess the clinical outcomes related to the lipid profile and safety profile of lomitapide in these populations.

## Methods

We performed this systematic review and meta-analysis following the Preferred Reporting Items for Systematic Reviews and Meta-analyses (PRISMA) guideline [16]. We registered it in PROSPERO (CRD420251016819).

### Sources of Data & Search Strategy

Until March 2025, we conducted systematic searches in the following databases: Scopus, PubMed, and Web of Science. We have included all the terms, keywords, and their synonyms for each database separately to improve the search results. Our search strategy was: (Lomitapide OR AEGR733 OR BMS 201038) AND (“Homozygous Familial Hypercholesterolemia” OR HoFH OR Hypercholesterolemia OR Hypercholesterolaemia OR"Elevated Cholesterol"OR"High Cholesterol Level").

### Eligibility Criteria

Our study inclusion criteria included the following:

1. We included observational studies and clinical trials conducted on human subjects, without any restrictions on language.
2. The studies focused on familial homozygous hypercholesterolemic (HoFH) patients at any age group, gender, or ethnicity, who intervened on using lomitapide to assess its efficacy and safety.
3. The studies were required to provide sufficient information to conduct qualitative and/or quantitative analysis.

Any study that did not meet the inclusion criteria described previously was excluded, as were case reports, review articles, case series, animal studies, protocols, conference abstracts, theses, and oral presentations.

### Selection Process

The screening process was performed blindly by two independent authors on Rayyan software [17]. Firstly, the duplicates were removed, and they began with title and abstract screening, then they screened the full texts, and they included and excluded the papers according to the PICO criteria. Any conflicts in the inclusion decision between the two authors were resolved by consultation with a third author.

### Data Extraction

Four independent authors made the data extraction in an online Excel sheet. The sheet was split into 3 sections: study characteristics (sample size, study design, population, intervention with doses, concomitant treatment, and conclusion), baseline characteristics of the population (age, gender, and BMI), and outcome measures. Our outcome measures included efficacy and safety outcomes. The efficacy outcomes were assessed as either absolute changes in concentration or percentage reductions from baseline For each included study, we extracted lipid profile outcomes at the 24–26 week timepoint wherever available, as this is the most commonly reported and clinically validated interval for assessing lomitapide efficacy. This time window reflects a stable treatment response and aligns with the primary endpoints in regulatory trials. If multiple timepoints were reported, we selected the closest available value to 24–26 weeks to maintain consistency across studies. These timepoints are detailed in Table 1. The primary efficacy outcomes included: total cholesterol (TC), triglycerides (TG), high-density lipoprotein cholesterol (HDL-C), and low-density lipoprotein cholesterol (LDL-C). These parameters are widely used in clinical practice and serve as standard markers for evaluating lipid-lowering therapies. The secondary efficacy outcomes included: non–high-density lipoprotein cholesterol (non–HDL-C), very-low-density lipoprotein cholesterol (VLDL-C), apolipoprotein A (apoA), apolipoprotein B (apoB), and lipoprotein(a) [Lp(a)]. These secondary measures provide additional insight into the effect of therapy on atherogenic lipoprotein particles and residual cardiovascular risk. Adverse events assessed in this analysis included a range of commonly reported treatment-related side effects. These comprised abdominal pain, nausea, vomiting, diarrhea, musculoskeletal symptoms, respiratory events, and infections. Additionally, we evaluated the incidence of adverse events leading to treatment discontinuation as a key safety outcome.

**Table 1:** Characteristics of the included studies

Study	Sample size	Study design	Population	Duration of treatment with Intervention doses	Concomitant treatment	Conclusion
Masana et al. 2024 [13]	43	Single-arm, open-label, phase 3 study	Pediatric patients diagnosed with Homozygous familial hypercholesterolemia	Lomitapide initial dose: 2 mg/day (ages 5–15) and 5 mg/day (ages 16–17), escalated over 24 weeks to a mean tolerated dose of 9.7 mg/day	All patients maintained stable lipid-lowering therapy (statins, ezetimibe, PCSK9 inhibitors, lipoprotein apheresis) 19 patients (44%) received lipoprotein apheresis	Mean LDL reduction: 53.5% (p < 0.0001) at 24 weeks 18 patients (42%) achieved LDL < 135 mg/dL, and 16 (37%) achieved LDL < 115 mg/dL Adverse effects: Mainly GI (diarrhea, abdominal pain)—Increased Liver enzymes (37%)
D'Erasmo et al. 2022 [21]	75	Multicenter retrospective observational study	Patients aged > 18 years diagnosed with homozygous familial hypercholesterolemia and have been receiving lomitapide for at least 1 month	Lomitapide: mean dose of 20 mg/day for 24 weeks	Concomitant lipid-lowering therapy (LLTs) included statins, ezetimibe, PCSK9i, fibrates, and lipoprotein apheresis	Lomitapide resulted in a significant and powerful reduction in serum cholesterol at a lower dose of 20 mg/day. A large proportion of patients were able to discontinue lipoprotein apheresis after the addition of lomitapide. Lomitapide has a favorable safety profile
Kolovou et al. 2020 [18]	12	Single-arm (pre-post) clinical trial	Patients aged 8–62 years with a clinical and genetic diagnosis of familial hypercholesterolemia	The mean dose of Lomitapide was 21.4 mg/day For nearly 20 weeks	Patients were maintained on a low-fat diet and statin therapy, in combination with ezetimibe and/or colesevelam, with or without lipoprotein apheresis performed every 7 to 15 days	The addition of Lomitapide therapy resulted in a significant reduction in LDL cholesterol by 57% compared to standard LLT alone and by 54% compared to LLT plus lipoprotein apheresis
Underberg et al. 2020 [23]	187	Multicenter, long-term, prospective, cohort	Adult patients ≥ 18 years with familial hypercholesterolemia receiving lomitapide	Lomitapide was administered at a median dose of 10 mg/day with a dosing range from 2.5–40 mg/day For 24 weeks	Lipoprotein apheresis, lipid-lowering drugs, or a combination of the two were used to treat the patients, based on their response to treatment and specific clinical needs	Lomitapide, as an adjunct to other lipid-lowering therapies, has a favourable long-term benefit-risk profile. LDL cholesterol targets have been reached with a 10 mg/day dose that is a quarter of the dose used in the phase 3 trial, contributing to improved tolerability
D'Erasmo et al. 2017 [20]	15	Multicenter retrospective observational study	Patients with genetically confirmed homozygous familial hypercholesterolemia	Lomitapide with a mean dose of 19 ± 13.3 mg/day For 32.3 ± 29.7 months	All patients were receiving standard lipid-lowering therapy (LLTs) (simvastatin, ezetimibe, rosuvastatin, and fenofibrate in different combinations) before adding lomitapide, and ten patients were receiving lipoprotein apheresis (LA) in addition to LLTs	Lomitapide resulted in a significant reduction of cholesterol levels in patients with homozygous familial hypercholesterolemia regardless of genotype. None of the patients had to stop medication because of liver or GI-related side effects. The most common GI-related side effect was diarrhea
Harada-Shiba et al. 2017 [22]	9	Single-arm, open-label, phase 3 study	Japanese adults with Homozygous familial hypercholesterolemia	Lomitapide [initial dose 5 mg/day, escalated to 60 mg/day based on tolerability. This was for 26 weeks	All patients maintained lipid-lowering therapy (statins, ezetimibe, probucol, colestilan, or ethyl eicosapentaenoic acid) 6 patients underwent Lipoprotein apheresis at baseline Diet restriction < 20% of total caloric intake from fat, with vitamin E and essential fatty acid supplementation	Lomitapide induced a significant reduction of LDL by 42% at 26 weeks (p < 0.0001) and 38% at 56 weeks (p = 0.0032), as well as non-HDL cholesterol, VLDL, triglycerides, and apolipoprotein B Most common adverse effects: GI symptoms—Liver enzyme elevation (in 3 patients)
Cuchel et al. 2013 [9]	29	Single-arm, open-label, phase 3 study	Adults with Homozygous familial hypercholesterolemia	Lomitapide [initial dose 5 mg/day, increased to max 60 mg/day (based on tolerability and safety)] For 26 weeks	All patients maintained lipid-lowering therapy (statins, ezetimibe, niacin, fibrates, or bile acid sequestrants), a low-fat diet, and dietary supplementation (vitamin E and essential fatty acids) 18 patients underwent LDL Apheresis	Lomitapide usage resulted in a significant reduction in LDL levels by 50% by 26 weeks & maintained at 78 weeks The most common adverse effects were GIT related
Cuchel et al. 2007 [19]	6	Open-label dose escalation study	Adults with genetically confirmed homozygous familial hypercholesterolemia	Lomitapide was escalated stepwise every four weeks to 0.03, 0.1, 0.3, and 1.0 mg/kg/day For 4 weeks	All lipid-lowering therapies, including apheresis, were stopped 4 weeks before treatment and remained discontinued throughout the study	LDL reduction by 50.9% at dose 1.0 mg/kg/day (p < 0.001) Total cholesterol was reduced by 58.4%, whilst TGA by 65.2% Adverse effects: Mild GI effects—Liver enzyme elevation (in 2 patients) [returned to baseline within 4–14 weeks after drug discontinuation

*LDL*: low-density lipoprotein cholesterol; *VLDL*: very-low-density lipoprotein cholesterol; *HDL*: high-density lipoprotein cholesterol; *LLT*: lipid-lowering therapy

### Risk of Bias and Quality Assessment

All included studies were appraised by four independent authors using the NIH Quality Assessment Tool for Before-After (Pre-Post) studies with no control group [18]. The tool comprises twelve items evaluating key aspects of study design, including the clarity of study objectives, appropriateness and transparency of participant selection, adequacy of sample size, and proper implementation of the intervention. It also considers whether outcomes were measured using valid and reliable tools, and whether appropriate statistical analyses were conducted, including pre- and post-intervention comparisons and handling of potential issues such as loss to follow-up. Based on these criteria, each study was rated as good, fair, or poor in overall quality.

### Statistical Analysis

We conducted our single meta-analysis using the (meta) package in RStudio with a random effects model [19]. The analysis utilized weighted mean changes from baseline along with standard deviations, and results were presented with 95% confidence intervals (CIs). For dichotomous outcomes, pooled estimates were calculated as proportions with corresponding 95% CIs. Heterogeneity across studies was assessed using the I^2^ statistic (Higgins score), where an I^2^ value ≥ 50% indicated substantial heterogeneity. A chi-square test P-value < 0.1 was considered to reflect significant heterogeneity.

## Results

### Literature Search Results

**Fig. 1:**
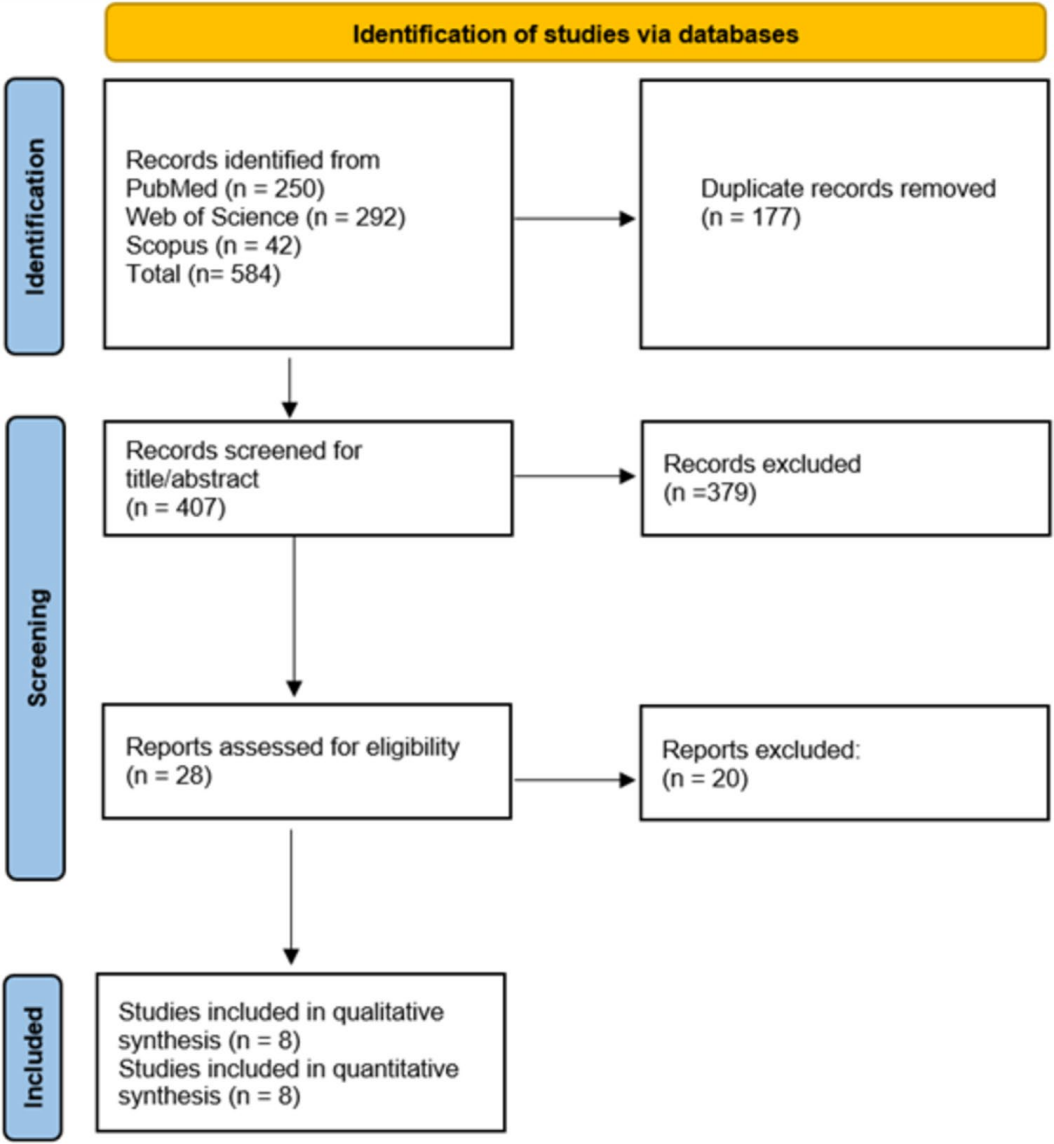
PRISMA flowchart of study selection

We identified a total of 584 records from the literature databases. After removing 177 duplicates, 407 articles were screened for eligibility based on title and abstract using Rayyan. This screening stage excluded 379 articles as they did not meet the inclusion criteria. Further, 28 records were assessed at the full-text level. Finally, eight studies were selected and included in the meta-analysis. The PRISMA flow diagram is shown in Fig. 1.

### Characteristics of the Included Studies

The studies included in our meta-analysis examined the use of lomitapide in patients with HoFH, with sample sizes ranging from 6 to 187 participants [11, 15, 20–25]. These studies involved both adult and pediatric populations, with mean ages spanning from 10.7 to 52.2 years. Baseline lipid profiles were markedly elevated, with LDL cholesterol levels ranging from approximately 199 mg/dL to over 800 mg/dL, and total cholesterol levels reaching up to 851 mg/dL. Triglycerides, non-HDL cholesterol, and VLDL levels were also consistently high across studies. BMI values were generally in the normal to overweight range (22–30 kg/m^2^), and xanthomas were frequently reported, with some studies documenting 100% prevalence among participants. All studies reported significant reductions in LDL cholesterol following Lomitapide treatment, often exceeding 50% from baseline. The drug was administered alongside conventional lipid-lowering therapies such as statins, ezetimibe, PCSK9 inhibitors, and lipoprotein apheresis, as well as dietary modifications. Many patients were able to reduce or discontinue apheresis following treatment. The most common adverse effects were gastrointestinal, including diarrhea and abdominal discomfort, along with occasional liver enzyme elevations. Summary of the included studies and baseline characteristics of the enrolled patients are represented in Tables 1 and 2, respectively.

**Table 2:** Baseline characteristics of the population

Study ID	Age, year, Mean (SD)	Gender, male, N (%)	BMI, (kg/m2)	Xanthoma, N (%)	TC (mg/dl)		LDL-C (mg/dl)		VLDL-C (mg/dl)		non-HDL-C (mg/dl)		TGA (mg/dl)		HDL-C (mg/dl)	
					N	Mean (SD)	N	Mean (SD)	N	Mean (SD)	N	Mean (SD)	N	Mean (SD)	N	Mean (SD)
Masana et al. 2024 [13]	10.7 (3.2)	19 (44%)	NA	NA	43	440.8 (210.2)	43	390.5 (189.5)	43	17 (7.1)	43	407.5 (202.8)	43	85.8 (60.2)	NA	NA
D'Erasmo et al. 2022 [21]	43.0 (19.0)	37 (49.3%)	24.9 (4.9)	51 (68.0%)	75	364.7 (173.1)	75	292.4 (161.2)	NA	NA	NA	NA	75	101.3 (52.9)	75	42.5 (12.9)
Kolovou et al. 2020 [18]	8–62*	8 (66.7%)	22.4 (3.6)	12 (100%)	12	895.75 (219.3)	12	804.5 (220.8)	NA	NA	NA	NA	12	162.75 (69.4)	12	36 (7.6)
Underberg et al. 2020 [23]	52.2 (15.3)	78 (41.7%)	30.4 (6.8)	NA	NA	NA	187	232 (94.98)	NA	NA	NA	NA	NA	NA	NA	NA
D'Erasmo et al. 2017 [20]	37.7 (13.5)	6 (40%)	23 (4.6)	13 (86.6%)	15	492.7 (201)	15	426 (204)	NA	NA	15	447.6 (204.1)	15	106.8 (36)	15	47.8 (14.9)
Harada-Shiba et al. 2017 [22]	50.3 (14.7)	5 (56%)	22.1 (4.3)	NA	9	279 (80)	9	199 (66)	9	25 (13)	9	228 (78)	9	135.5 (70.9)	9	50 (10)
Cuchel et al. 2013 [9]	30.7 (12.1)	16 (55.2%)	25.9 (5.4)	24 (83%)	29	428.7 (135.5)	29	336.8 (112.2)	29	19.3 (11.6)	29	386.7 (131.6)	29	117.4 (55.4)	29	42.9 (11.7)
Cuchel et al. 2007 [19]	25.7 (9.3)	3 (50%)	24.8 (4.4)	NA	6	851 (198)	6	614 (105.8)	6	209 (202)	NA	NA	6	283 (190)	6	*26 (6)*

*LDL-C*: low-density lipoprotein cholesterol; *VLDL-C*: very-low-density lipoprotein cholesterol; *HDL-C*: high-density lipoprotein cholesterol; *TC*: Total cholesterol; *TG*: Triglycerides; *SD*: Standard deviation; *BMI*: Body mass index; *N*: Number; *NA*: Not available, * range

### Quality Assessment

The quality assessment of the included studies, based on the NIH tool, showed that most were of fair quality, with scores ranging from 7 to 8. One study (Masana et al. 2024) [15] was rated as good, while two studies (Kolovou et al. 2020 [20] and Underberg et al. 2020 [25]) were rated poor due to limited reporting and methodological weaknesses. Overall, the studies demonstrated acceptable methodological quality, though some design limitations may impact the strength of the findings. The complete quality assessment table for the included studies, with further details, is provided in Supplementary Table 1.

### Efficacy Endpoints

#### Primary Efficacy Outcomes [TC, HDL-C, LDL-C, and TG]

**Fig. 2:**
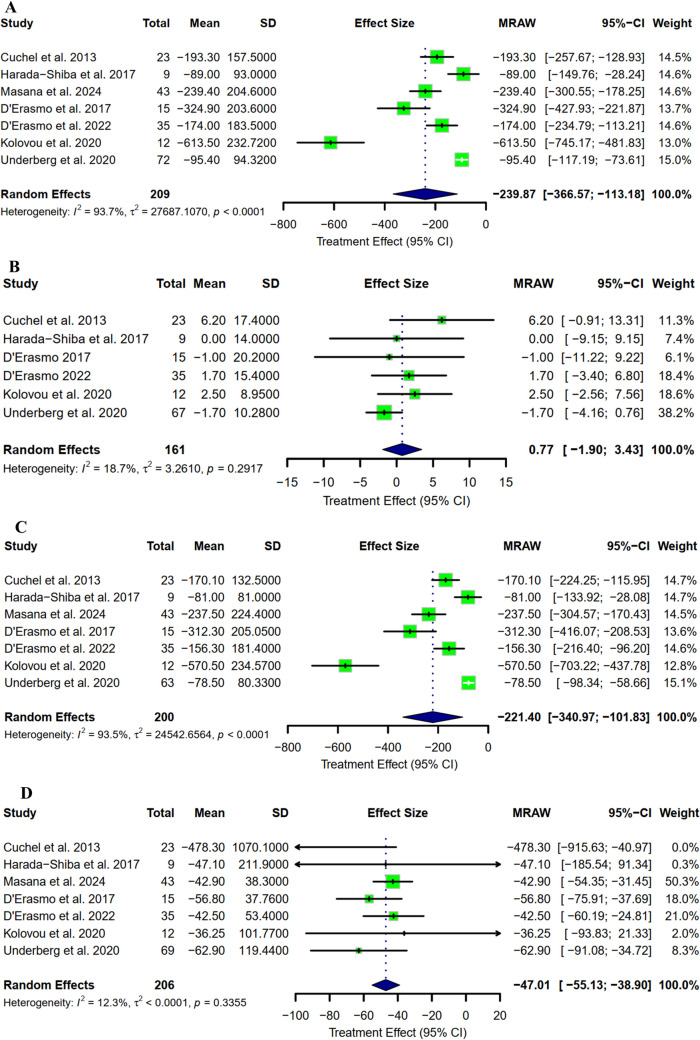
Mean change from baseline in terms of **A)** Total cholesterol; **B)** HDL-C; **C)** LDL-C; **D)** TG

**Fig. 3:**
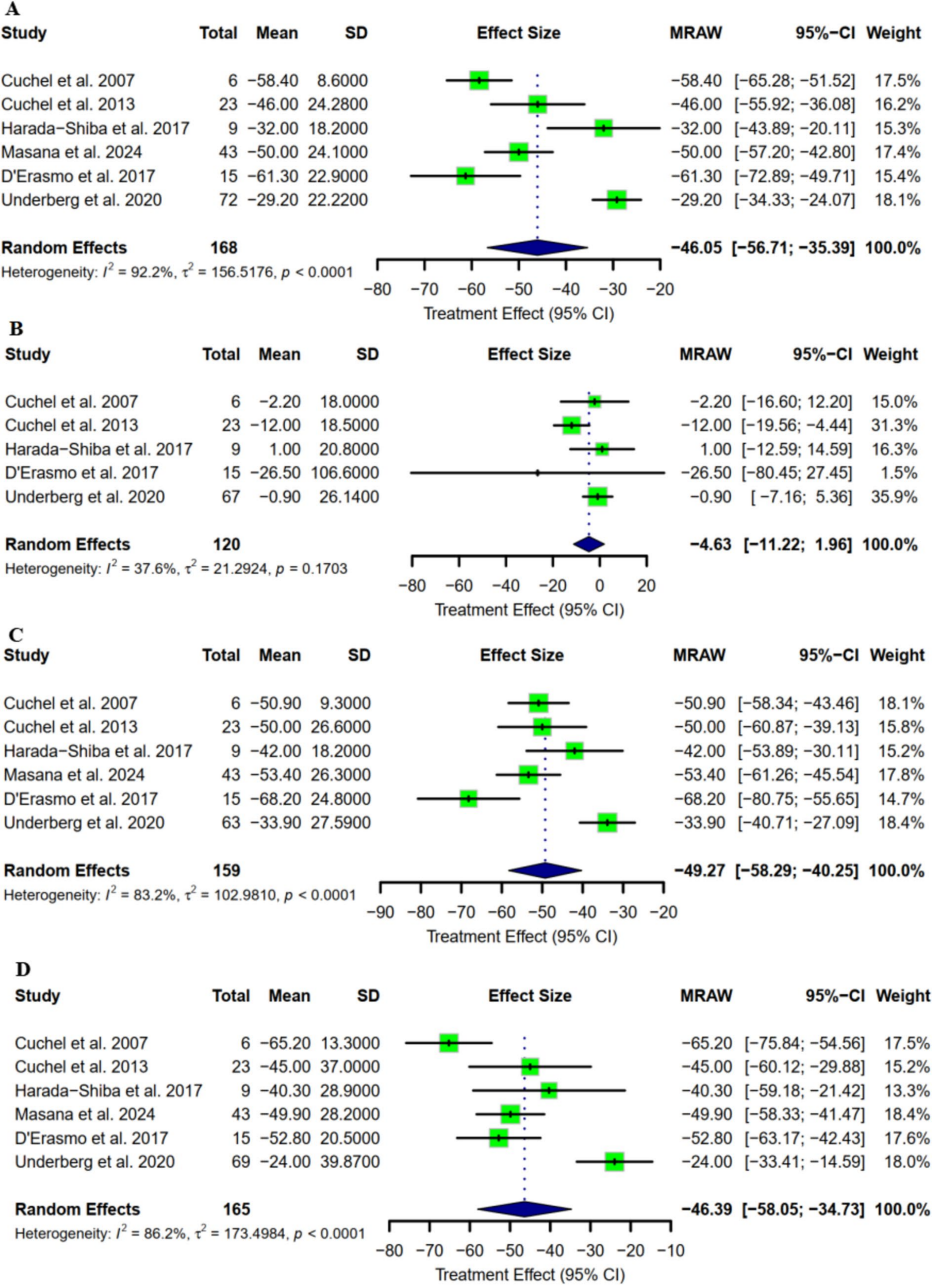
Mean percentage change from baseline in terms of **A)** Total cholesterol; **B)** HDL-C; **C)** LDL-C; D**)** TG

Seven studies with a combined total of 209 participants reported a change from baseline in TC level. The pooled mean change was −239.87 mg/dl (95% CI: [−366.57 to −113.18] as represented in Fig. 2A). The mean percentage reduction was −46.05 (95% CI: [−56.71 to −35.39] as represented in Fig. 3A). Regarding HDL-C, the mean change was reported in six studies, with a pooled mean change of 0.77 mg/dl (95% CI: [−1.90 to 3.43] as represented in Fig. 2B). The mean percentage change was −4.63 (95% CI: [−11.22 to 1.96] as represented in Fig. 3B). LDL-C mean concentration change from baseline was reported in seven studies, with a pooled mean of –221.40 mg/dl (95% CI: [−340.97 to −101.83] as represented in Fig. 2C). The pooled LDL-C mean percentage reduction was −49.27 (95% CI: [−58.29 to −40.25] as represented in Fig. 3C). Triglycerides concentration change from baseline has been reported in seven studies, with a pooled mean change of −47.01 mg/dl (95% CI: [−55.13 to −38.90] (as represented in Fig. 2D). The pooled percentage reduction in TGA was −46.39 mg/dl (95% CI: [−58.05 to −34.73] as represented in Fig. 3D).

#### Secondary Efficacy Outcomes [Lp(a), apoA, apoB, VLDL-C, and non–HDL-C]

**Fig. 4:**
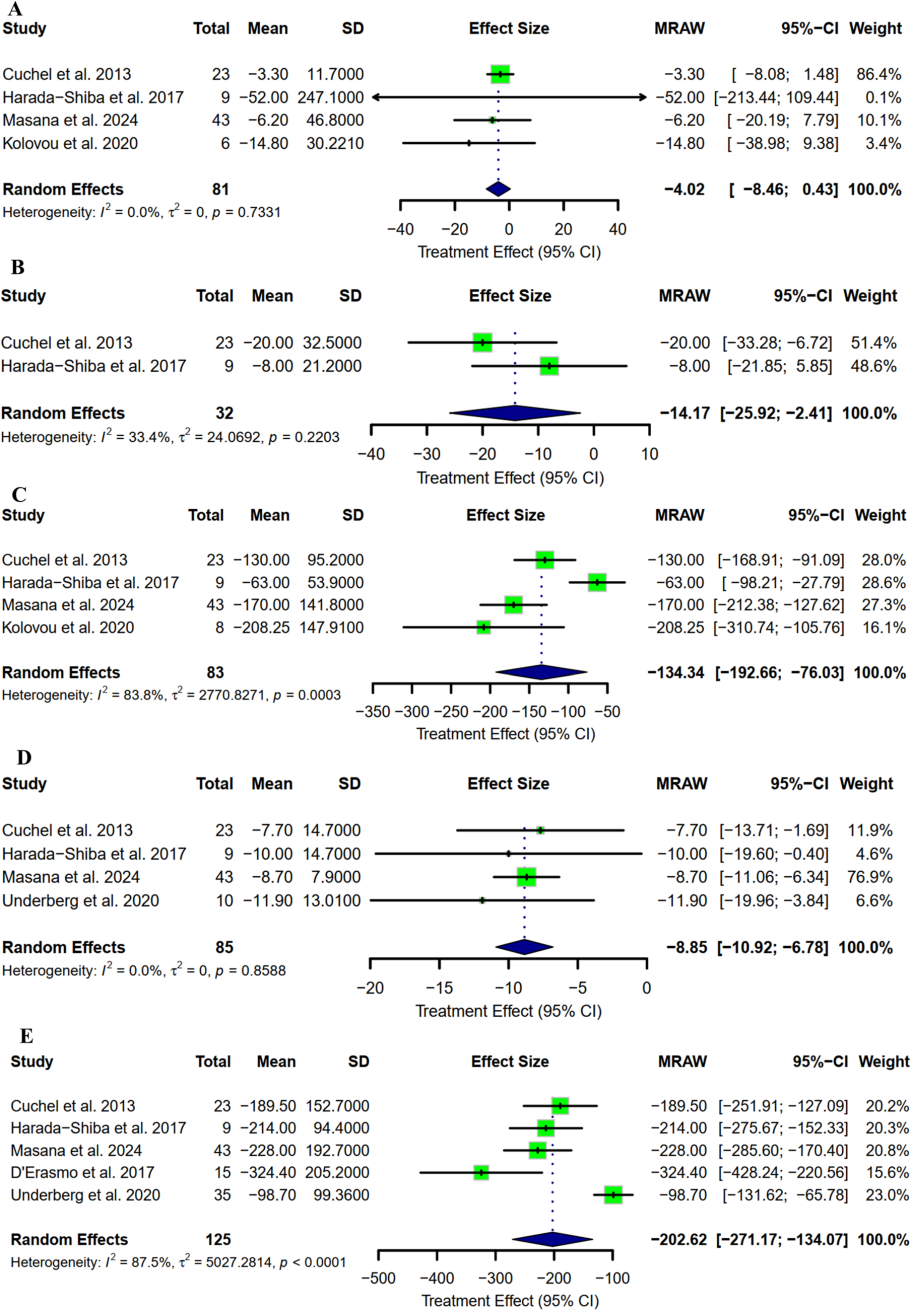
Mean change from baseline in terms of **A)** Lp(a); **B)** apoA; C**)** apoB; **D)** VLDL-C; **E)** non–HDL-C

**Fig. 5:**
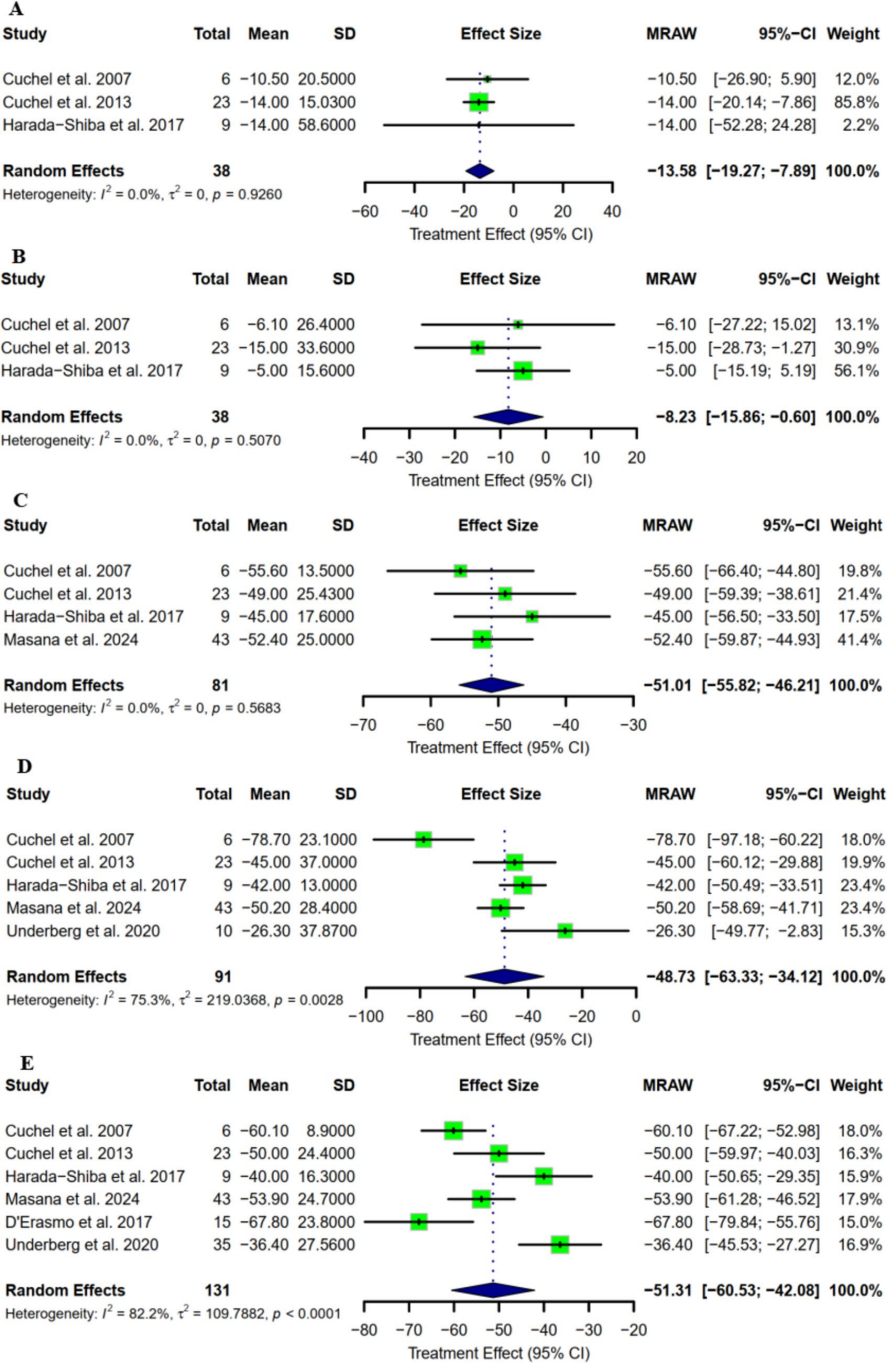
Mean percentage change from baseline in terms of **A)** Lp(a); **B)** apoA; C**)** apoB; **D)** VLDL-C; **E)** non–HDL-C

Regarding lipoprotein A, the mean reduction from baseline was −4.02 mg/dl (95% CI: [−8.46 to 0.43] as represented in Fig. 4A), with a percentage reduction of −13.58 (95% CI: [−19.27 to −7.89] as represented in Fig. 5A). Moreover, the pooled mean change from baseline in apolipoprotein A1 was −14.17 mg/dl (95% CI: [−25.92 to −2.41] as represented in Fig. 4B), and a pooled percentage reduction of −8.23 (95% CI: [−15.86 to −0.60] as represented in Fig. 5B). Apolipoprotein B pooled mean change from baseline was −134.34 mg/dl (95% CI: [−192.66 to −76.03]as represented in Fig. 4C), with a percentage reduction of −51.01 (95% CI: [−55.82 to −46.21] as represented in Fig. 5C). VLDL-C showed a decreasing trend with a mean change from baseline of −8.85 mg/dl (95% CI: [−10.92 to −6.78] as represented in Fig. 4D), and a percentage reduction of −48.73 (95% CI: [−63.33 to −34.12] as represented in Fig. 5D). With respect to non-HDL-C, the pooled mean change from baseline reported by five studies was −202.62 mg/dl (95% CI: [−271.17 to −134.07] as represented in Fig. 4E), and the mean percentage reduction was −51.31 (95% CI: [−60.53 to −42.08] as represented in Fig. 5E).

### Adverse Events

**Fig. 6:**
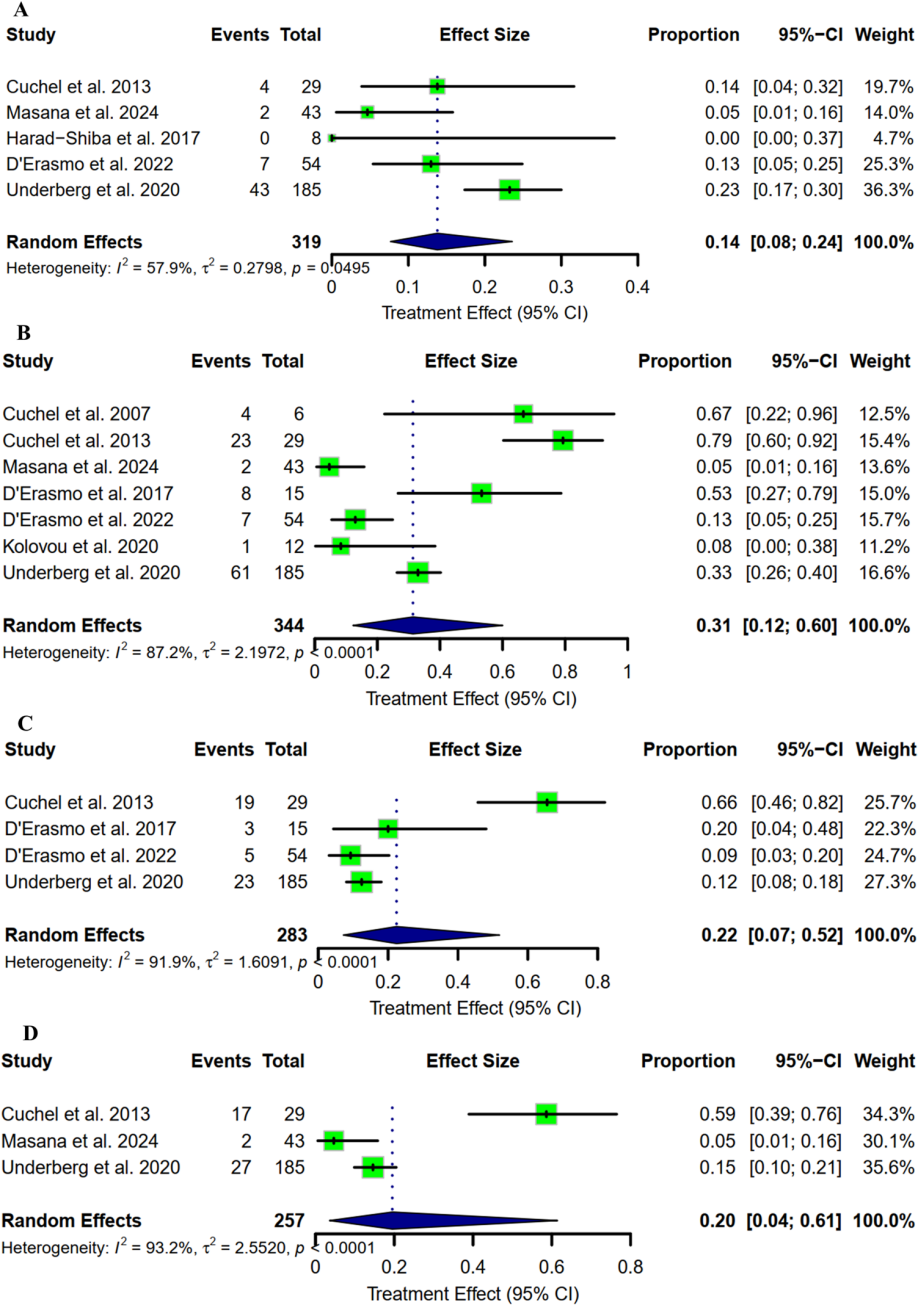
**A)** Side effects-related discontinuations; **B)** Diarrhea; **C)** Nausea; **D)** Infections

The adverse events reported by the included studies were, in general, acceptable. However, our analysis showed a pooled proportion of 14% (95% CI: [8% to 24%]) of adverse events leading to discontinuations (Fig. 6A). The most reported and noticeable side effects were diarrhea, nausea, and infections with a pooled proportion of 31%, 22%, 20%, respectively (Fig. 6B, C, D respectively). Further side effects, including musculoskeletal, respiratory, vomiting, and abdominal pain, were not significant, as represented in Supplementary Fig. 1.

## Discussion

This meta-analysis rigorously encompassed up-to-date literature and pooled eight studies with a total of 376 patients to provide robust evidence demonstrating that lomitapide considerably improved lipid levels in the homozygous familial hypercholesterolemic patient with HoFH. Our study demonstrated a noticeable LDL-C reduction of 49.27%, accompanied by a decrease in total cholesterol (TC) (−46.05%) and non–HDL-C (−51.31%). In addition, apolipoprotein B (apoB) was reduced by 51.01%, reflecting Lomitapide's capacity to reduce atherogenic lipoproteins, significant drivers of cardiovascular risk [26]. Conversely, HDL-C levels remained relatively unchanged, with a minimal reduction of 4.63%. TG and VLDL-C, however, experienced considerable reductions of 46.39% and 48.73%, respectively. These reductions are of clinical importance as lowered triglycerides have been found to be associated with lowered risk for large vascular events, even when risk adjustment is performed for LDL-C reduction in HoFH patients [27]. In terms of safety, side effects from the included trials were generally tolerable. However, 14% of patients withdrew from the trials due to side effects. The most common adverse reactions were diarrhea (31%), nausea (22%), and infections (20%).

Before the introduction of contemporary lipid-lowering therapies, patients with HoFH often presented with extremely elevated LDL-C levels, ranging from 13–27 mmol/L (500–1000 mg/dL) [1, 28, 29]. While statins remain a cornerstone of therapy, their effectiveness in HoFH is often limited due to insufficient LDL receptor activity [30]. Similarly, PCSK9 inhibitors, though revolutionary in general hyperlipidemia management, show reduced efficacy in HoFH, with only 23% LDL-C reduction over 48 weeks [31]. Given this context, the LDL-C reduction achieved with Lomitapide may lead to improved life expectancy and lower cardiovascular event rates in HoFH patients [32, 33]. While lipoprotein apheresis is another treatment option capable of acutely reducing LDL-C by over 70%, its effects are short-lived [34]. In contrast, Lomitapide offers sustained long-term control of LDL-C levels [35]

Regarding safety, our findings of 14% discontinuation due to adverse events appear lower than previously reported in early trials [36], where gastrointestinal disturbances were more prevalent (e.g., diarrhea in 79%, nausea in 65%, and GI symptoms overall in 93%). However, findings from longer-term real-world studies, consistent with clinical trial experience, suggest that Lomitapide is associated with a risk of hepatic steatosis. Liver imaging has demonstrated a moderate increase in hepatic fat content, typically accompanied by normal liver stiffness [37]. In pediatric populations, one study reported that transient elevations in liver function tests, exceeding three times the upper limit of normal, may occur. These changes are generally manageable through temporary dose reduction, followed by gradual re-titration to the previous dose [38]. Notably, hepatic steatosis may develop with or without concurrent elevations in liver enzymes, particularly transaminases [39]. Encouragingly, emerging long-term real-world evidence shows that Lomitapide can maintain significant LDL-C reductions for up to seven years without discontinuation, supporting its potential as a durable treatment option [40]. Nevertheless, further studies are needed to evaluate long-term adherence, explicitly reporting the duration of therapy and reasons for discontinuation, if any, to better inform clinical decision-making. Additionally, the Homozygous Familial Hypercholesterolemia International Clinical Collaborators (HICC) consortium, which was not included in our analysis due to the case-based nature of reporting, provided perspectives of Lomitapide use in real-world settings in a multinational cohort. While the consortium findings are not limited to Lomitapide, it did highlight the increasing utilization of Lomitapide, most often in combination with other therapies, including statins, ezetimibe and PCSK9 Inhibitors, with higher rates of effectiveness than conventional treatments alone. Despite this, Tromp et al., reported that out of 751 patients across 38 countries, only 14.7% were successfully treated with Lomitapide, with minimal to no usage in low- and middle-income countries, which suggests geographic disparities leading to significant gaps in drug usage due to global accessibility, as a result of high cost, lack of availability or guideline enrolment and clinician unfamiliarity in the global south regions [2]. Furthermore, in another report by Mulder and Tromp et al., sex-based disparities were found, with female patients having a more notably delayed diagnosis, which contributed to delaying the initiation of appropriate treatment, including Lomitapide, and affecting these patient outcomes [41].

This single-arm meta-analysis, to the best of the authors'knowledge, is the first to evaluate the efficacy and safety of lomitapide in HoFH patients. It encompasses real-world and clinical trial evidence across adult and pediatric patients, and this provides a snapshot of lomitapide's impact. Strengths of the analysis are the use of standardized outcome measures, rigorous methodology, and inclusion of studies with varying populations and treatment histories. However, there is no control group to be drawn upon, and causality establishment is restricted; the overall quality of the included studies was mostly fair, but some were rated as poor. The other restrictions are small sample sizes and variable follow-up reporting. Future research needs to focus on well-executed randomized controlled trials of longer duration with larger and more representative cohorts. Furthermore, ongoing publication of long-term efficacy and adverse events, particularly hepatic-associated ones, will be key to guiding clinical decision-making and optimizing the therapeutic use of lomitapide.

## Conclusion

This meta-analysis showed lomitapide as a potent lipid-lowering therapy for the treatment of HoFH patients with a near-50% reduction in LDL-C concentrations, as well as significant reductions in other atherogenic lipoproteins such as total cholesterol, triglycerides, non-HDL-C, and apoB. Lomitapide's safety profile, with the attendant side effect of gastrointestinal distress, was generally acceptable, with a rate of discontinuation of 14%. These findings indicate the clinical potential of lomitapide when standard therapy with statins and PCSK9 inhibitors is unable to be effective due to impaired activity of the LDL receptors. While promising, this review faults the requirement for more robust randomized controlled trials with larger and more diverse study populations, longer follow-up, and systematic reporting of hepatic and adherence outcomes.

## Supplementary Information

Below is the link to the electronic supplementary material.

Supplementary file1 (DOCX 861 KB)

## Data Availability

All data generated or analyzed during this study are included in this published article [and its supplementary information files].

## Abbreviations

HoFH: Homozygous Familial Hypercholesterolemia
LDL-C: Low-density lipoprotein cholesterol
MTP: Microsomal triglyceride transfer protein
PRISMA: The Preferred Reporting Items for Systematic Reviews and Meta-analyses
TC: Total cholesterol
TGA: Triglycerides
HDL-C: High-density lipoprotein cholesterol
VLDL-C: Very low-density lipoprotein cholesterol
apoA: Apolipoprotein A
apoB: Apolipoprotein B
Lp(a)]: Lipoprotein(a)
95% CIs: 95% Confidence intervals

## Code availability

RStudio (“meta” package).
