# Supplementary material for: Lomitapide, a Microsomal Triglyceride Transfer Protein Inhibitor, in Homozygous Familial Hypercholesterolemia: A Systematic Review and Meta-Analysis of Efficacy and Safety
Source: Cardiovasc Drugs Ther. 2025 Aug 26;40(3):953–69. doi: 10.1007/s10557-025-07764-4 (PMC13171640; doi:10.1007/s10557-025-07764-4)
Supplement: Supplementary file 1 — Supplementary file1 (DOCX 861 KB) [file 10557_2025_7764_MOESM1_ESM.docx]

| Study ID | Q1 | Q2 | Q3  **Supplementary table 1:** NIH quality assessment of the included studies | Q4 | Q5 | Q6 | Q7 | Q8 | Q9 | Q10 | Q11 | Q12 | Total |
| --- | --- | --- | --- | --- | --- | --- | --- | --- | --- | --- | --- | --- | --- |
| Cuchel et al. 2013 | Y | Y | Y | Y | N | Y | Y | N | N | Y | Y | NA | 8 FAIR |
| Harada-Shiba et al. 2017 | Y | N | Y | Y | N | Y | Y | N | Y | Y | NR | NA | 7 FAIR |
| Masana et al. 2024 | Y | Y | Y | Y | Y | Y | Y | N | Y | Y | Y | NA | 10 GOOD |
| Cuchel et al 2007 | Y | Y | N | Y | N | Y | Y | NR | Y | Y | Y | NA | 8 FAIR |
| D'Erasmo 2017 | Y | Y | N | Y | NR | Y | Y | N | Y | Y | Y | NA | 8  FAIR |
| D'Erasmo 2022 | Y | Y | N | Y | NR | Y | Y | N | Y | Y | Y | NA | 8  FAIR |
| Kolovou 2020 | Y | N | Y | NR | NR | N | Y | NR | Y | Y | N | NA | 5  Poor |
| Underberg 2020 | Y | Y | Y | NR | NR | N | NR | N | Y | N | Y | NA | 5  Poor |

Y=yes; N= no; NA= not applicable; NR= not reported

Good=10-12, Fair= 6-9, Poor= 0-5

1. Was the study question or objective clearly stated?

2. Were eligibility/selection criteria for the study population prespecified and clearly described?

3. Were the participants in the study representative of those who would be eligible for the test/service/intervention in the general or clinical population of interest?

4. Were all eligible participants that met the prespecified entry criteria enrolled?

5. Was the sample size sufficiently large to provide confidence in the findings?

6. Was the test/service/intervention clearly described and delivered consistently across the study population?

7. Were the outcome measures prespecified, clearly defined, valid, reliable, and assessed consistently across all study participants?

8. Were the people assessing the outcomes blinded to the participants' exposures/interventions?

9. Was the loss to follow-up after baseline 20% or less? Were those lost to follow-up accounted for in the analysis?

10. Did the statistical methods examine changes in outcome measures from before to after the intervention? Were statistical tests done that provided p values for the pre-to-post changes?

11. Were outcome measures of interest taken multiple times before the intervention and multiple times after the intervention (i.e., did they use an interrupted time-series design)?

12. If the intervention was conducted at a group level (e.g., a whole hospital, a community, etc.) did the statistical analysis take into account the use of individual-level data to determine effects at the group level


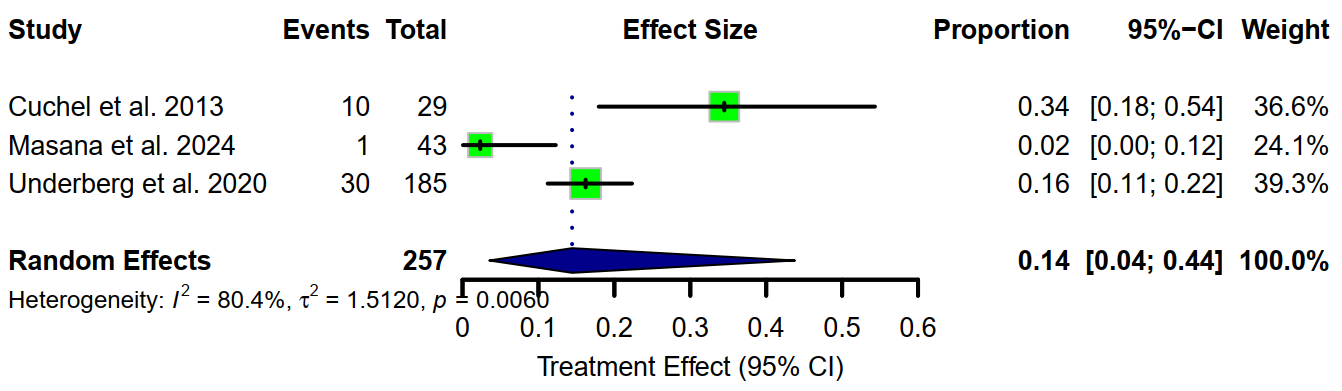

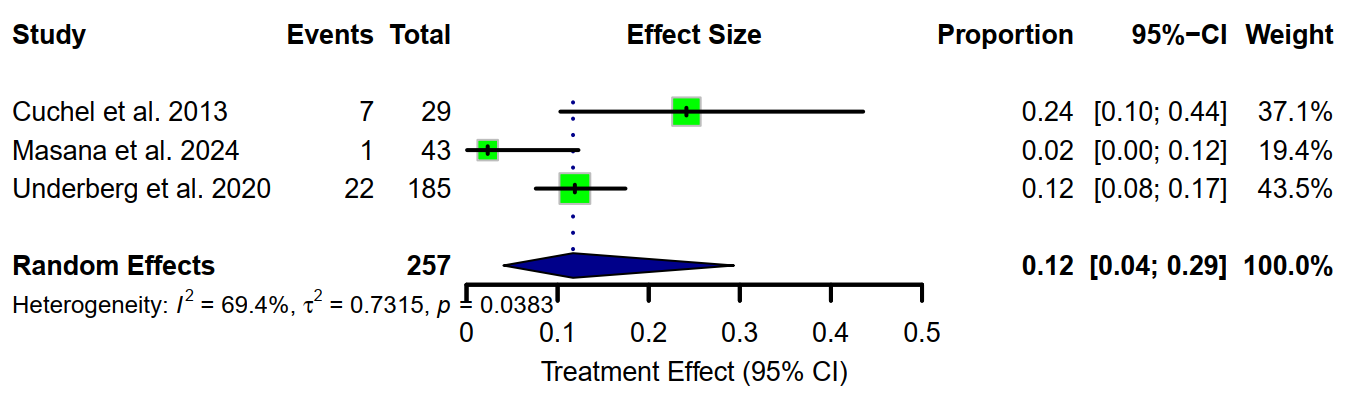

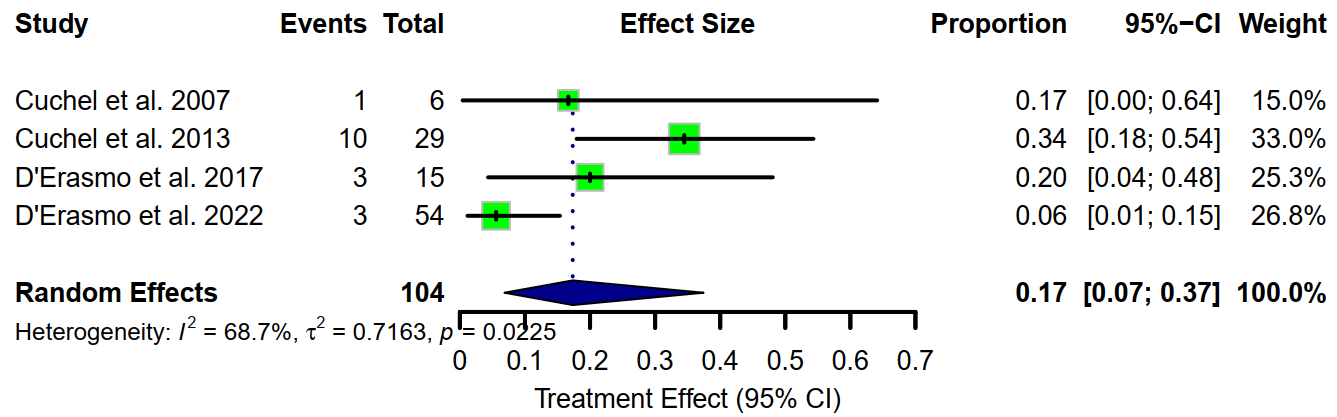

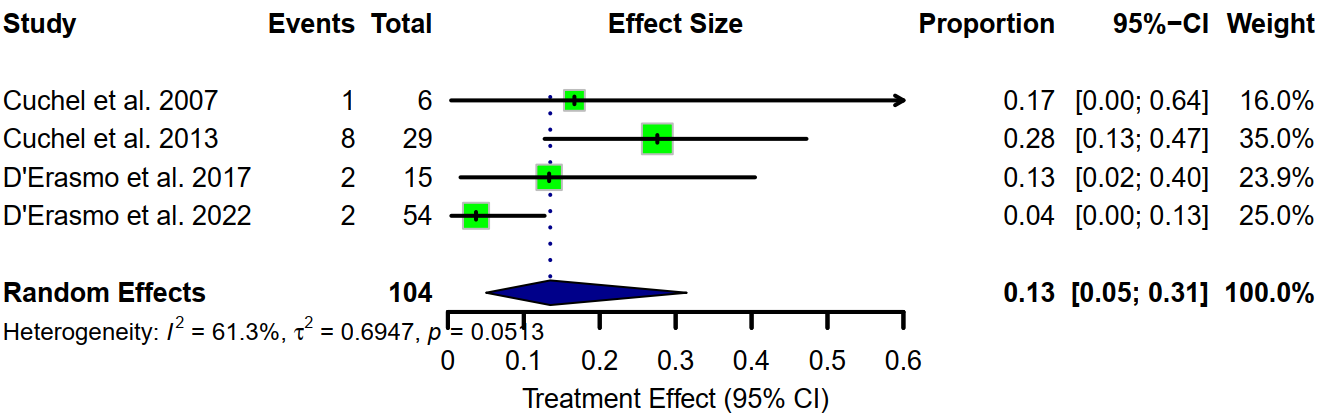


**A**

**B**

**C**

**D**

**Supplementary figure 1: A)** Musculoskeletal side effects; **B)** Respiratory side effects; **C)** vomiting; **D)** Abdominal pain
